# A Novel Improved Whale Optimization Algorithm-Based Multi-Scale Fusion Attention Enhanced SwinIR Model for Super-Resolution and Recognition of Text Images on Electrophoretic Displays

**DOI:** 10.3390/biomimetics11030195

**Published:** 2026-03-06

**Authors:** Xin Xiong, Zikang Feng, Peng Li, Xi Hu, Jiyan Liu, Xueqing Liu

**Affiliations:** 1 Key Laboratory of Optoelectronic Chemical Materials and Devices, Ministry of Education, School of Optoelectronic Materials & Technology, Jianghan University, Wuhan 430056, China; scysxiongxin@hotmail.com (X.X.); fengzikang054@gmail.com (Z.F.); 2 Flexible Display Materials and Technology Co-Innovation Center of Hubei Province, Jianghan University, Wuhan 430056, China; 3 School of Artificial Intelligence, Jianghan University, Wuhan 430056, China; huxi@jhun.edu.cn; 4 School of Intelligent Manufacturing, Jianghan University, Wuhan 430056, China; lpeng520@jhun.edu.cn

**Keywords:** Electrophoretic Displays (EPDs), Improved Whale Optimization Algorithm-based Multi-scale Fusion Attention enhanced SwinIR (IWOA-MFA-SwinIR) model, super-resolution and recognition of text images

## Abstract

Electrophoretic Displays (EPDs) are widely adopted in e-readers and portable devices due to their ultra-low power consumption and eye-friendly reflective characteristics. However, inherent hardware limitations, such as low resolution, slow response speed, and display degradation, frequently result in blurred strokes and degraded text readability. While traditional driving waveform optimizations can mitigate these issues, they are device-dependent and require extensive manual calibration. To address these challenges, this paper proposes an Improved Whale Optimization Algorithm-based Multi-scale Fusion Attention-enhanced SwinIR (IWOA-MFA-SwinIR) model for super-resolution and recognition of text images on EPDs. Structurally, the model incorporates a multi-scale fused attention (MFA) module that synergistically integrates channel, spatial, and gated attention mechanisms to precisely capture high-frequency text details while suppressing background noise within the SwinIR architecture. Furthermore, to enhance model robustness and eliminate manual tuning, an Improved Whale Optimization Algorithm (IWOA) is employed to adaptively optimize critical hyperparameters, including embedding dimension (*d*), attention head count (*h*), learning rate (lr), and dimensionality reduction coefficient (*r*). Experiments conducted on the TextZoom and EPD datasets demonstrate that the proposed model achieves state-of-the-art performance. In the ablation study, it attains a Peak Signal-to-Noise Ratio (PSNR) of 24.406, a Structural Similarity Index (SSIM) of 0.8837, and a Character Recognition Accuracy (CRA) of 89.81%. In the comparative evaluation, the proposed model consistently outperforms the second-best comparison model across three difficulty levels, yielding approximately a 1% improvement in PSNR, a 0.8% improvement in SSIM, and an 8% improvement in CRA. This confirms the proposed model’s superiority over mainstream comparative models in restoring text fidelity and improving recognition rates.

## 1. Introduction

Nowadays, electronic paper (e-paper), with its four differentiated advantages that include reflective eye-friendly viewing, ultra-low power consumption, flexibility, and legibility under sunlight, is promptly penetrating through the six fields of price tag, office, education, transportation, and medical logistics [1,2,3]. Taking e-paper applications in education as an instance, Electrophoretic Displays (EPDs) [4,5], as the display device of e-paper, unlike self-luminous Liquid Crystal Displays (LCDs) or Organic Light-Emitting Diodes (OLEDs), are capable of reflecting ambient light as the light source rather than high-energy blue light to realize near-zero radiation and hold static text images without constant refresh, of which the rapid development and the wide promotion are beneficial for reducing the incidence of myopia after the large-scale replacement of primary and secondary educational scenes. Display technology of EPDs has the characteristics of ultra-low power consumption and comfortable viewing in outdoor light environments, making it an ideal carrier for portable and wearable flexible display terminals [6]. According to data from the National Health Commission [7], the overall myopia rate among children and adolescents in China is 52.7%, with a myopia rate of 35.6% among primary school students, 71.1% among junior high school students, and 80.5% among high school students. It becomes a public health issue that may affect the quality of the country’s population, economic competitiveness, and even national defense security [8].

Hence, it is imperative to advance the display technology of EPDs, whose flexibility has attracted extensive attention in recent years due to their bistable, reflective, ultra-low-power characteristics, and environmental friendliness, making them particularly suitable for text-dominant applications such as electronic readers, educational devices, and electronic shelf labels due to their appearance similar to paper, low power consumption, environmental friendliness, and other advantages [9,10]. Unlike emissive displays, EPDs rely on the electrophoretic motion of charged pigment particles to form text images, which enable paper-like readability and long-term static display without continuous power consumption [11].

However, the text visual quality of EPDs is fundamentally constrained by their electro-optical driving mechanisms, which may seriously affect text readability at the text image level [6]. It poses significant challenges for super-resolution and recognition of text images on EPDs. Limited grayscale capability [12], slow particle response [6], and strong history dependence [13] often result in blurred edges, broken strokes, and low effective resolution and contrast, which severely degrade the readability of small-size characters and compromise textual structural integrity essential for accurate recognition.

To address these limitations, recent research has increasingly tailored text image super-resolution algorithms on EPDs. Traditional approaches focused on designing systematic approaches to driving waveforms for further improving various properties on EPDs, such as reducing the driving time [14,15], enhancing the response latency [16], reducing the ghost image [17], and so forth.

Moreover, learning-based artificial intelligence (AI) approaches have recently been introduced into the EPD domain. In particular, Convolutional Neural Networks (CNNs) have been applied to tasks such as text image recognition, waveform design, and image-quality enhancement, indicating that data-driven models can capture complex EPD degradation characteristics that are difficult to formulate analytically [18]. This line of work marks an important shift from rule-based waveform engineering to learning-based modeling and correction of image super-resolutions on EPDs. However, CNNs are inherently limited in modeling long-range dependencies and global structural information. To address this, Swin Transformer adopts window-based self-attention with a shifted-window scheme, enabling global dependency modeling while maintaining efficient local computation [19]. Building on Swin Transformer, the SwinIR model has been proposed for image restoration, offering a better trade-off between restoration performance and computational efficiency.

Despite the better effectiveness of the above approaches for EPDs, there still exists an urgent problem: How to enhance the super-resolution and recognition of text images on EPDs, mainly because particles tend to aggregate in solution due to their high specific density and high surface energy, which leads to limited low resolution, slow response speed, and inherent display limitations. Therefore, there are certain limitations on the super-resolution and recognition of text images on EPDs.

(a) Traditional approaches require hardware-specific empirical calibration and primarily modulate particle dynamics rather than visual information fidelity; even with carefully optimized waveforms, text images rendered on EPDs often remain visually degraded, which may be unsuitable for text super-resolution and recognition on EPDs due to limited robustness to structural details and vulnerability to background noise.

(b) Existing learning-based AI approaches still focus primarily on ghost image detection or waveform optimization rather than intrinsic text image enhancement, which demonstrates insufficient capability to support the model’s robustness and generalizability, particularly in complex text image scenarios.

(c) Existing learning-based EPD approaches still focus primarily on artifact detection or waveform selection, rather than directly enhancing the displayed text image itself. In particular, the semantic structure of text, such as character strokes and glyph consistency, is rarely exploited, despite being crucial for text readability. General-purpose text image enhancement or super-resolution models are also insufficient for this task, as they emphasize low-level visual fidelity while neglecting semantic constraints, often resulting in distorted characters or ambiguous strokes.

To leverage the advantages of both categories, this paper proposes a novel Improved Whale Optimization Algorithm-based Multi-scale Fusion Attention enhanced SwinIR (IWOA-MFA-SwinIR) model to enhance the super-resolution and recognition of text images on EPDs, which is able to improve text readability at the text image level. This paper presents the MFA-SwinIR model, which enhances the original SwinIR model through the integration of channel, spatial, and gated attention mechanisms coupled with multi-scale feature fusion to enable precise text edge modeling and background noise suppression. Moreover, the incorporation of the IWOA for automatic hyperparameter search significantly strengthens model robustness and generalization, delivering state-of-the-art reconstruction and recognition performance on TextZoom and electronic paper benchmarks.

The major contributions of our study are listed as follows:

(a) To address the visual degradation problem of text images rendered on EPDs, we leverage artificial intelligence from a computer vision perspective, treating EPD text super-resolution as a perceptual image reconstruction problem rather than a hardware modulation task. Specifically, based on the original SwinIR model, we tailor a modularized multi-scale fused attention-enhanced SwinIR architecture by introducing channel attention, spatial attention, and gated attention mechanisms to solve the problems of limited robustness to text image structural details and strong background noise interference. This enables our algorithm to directly learn the mapping from low-quality to high-quality text image rendered on EPDs, which achieves precise modeling of character edges, effective suppression of redundant information, and further optimization of visual information fidelity without hardware-dependent calibration.

(b) To better support the model’s robustness and generalizability, a multi-scale feature fusion strategy has been designed to enhance the text restoration capability in complex scenarios. To fully utilize the features at different Transformer levels, this paper proposes a multi-scale fused attention (MFA) module that adaptively weights and fuses shallow texture information with deep semantic expressions, thereby significantly enhancing the robustness and generalization performance of the model, particularly in complex text image scenarios.

(c) To solve the problem of traditional WOA easily falling into local optima, this paper proposes an Improved Whale Optimization Algorithm (IWOA) incorporating Sobol sequence-based population initialization, nonlinear convergence factor adjustment, and enhanced boundary handling mechanisms. The IWOA is subsequently employed to optimize four critical hyperparameters of the MFA-SwinIR model—embedding dimension (*d*), attention head count (*h*), learning rate (lr), and dimensionality reduction coefficient (*r*). Experimental validation on the TextZoom and EPDs datasets demonstrates that the optimized model achieves superior Peak Signal-to-Noise Ratio (PSNR), Structural Similarity Index (SSIM), and Character Recognition Accuracy (CRA), which can verify the effectiveness of our proposed IWOA-MFA-SwinIR model.

The rest of this paper is arranged as follows: Section 2 presents a brief overview of related works that consist of driving waveform design, AI technology for enhancing image resolution on EPDs, and meta-heuristic optimization algorithms applied for improving AI models. Section 3 indicates a series of improvements on basic AI attention modules and traditional WOA. In Section 4, this paper proposes a novel IWOA-MFA-SwinIR model on EPDs to enhance text readability at the text image level without modifying display hardware or driving waveforms. Experimental simulations are conducted in Section 5 to comprehensively evaluate the performance of the proposed IWOA-MFA-SwinIR model on EPDs, which contains an ablation experiment and a comparative experiment. Section 6 presents the conclusions and directions for future works.

## 2. Related Works

EPDs have been widely studied due to their ultra-low power consumption and paper-like visual characteristics. However, inherent physical limitations of electrophoretic particles, such as slow response speed, particle hysteresis, and history-dependent behavior, often lead to generating worse text image quality on EPDs that mainly manifest as ghost images, low contrast, and blurred edges, especially for text-dominant content. As a result, improving text image quality on EPDs has long been a core research topic.

### 2.1. Driving Waveform Design

The EPDs technology has advanced rapidly and profoundly since the 1970s [4]. Among various implementations, micro-encapsulated EPD has emerged as a dominant approach in this field. In such systems, charged particle suspensions are sealed within numerous microscopic capsules, with particle motion precisely controlled by the voltage differential applied between pixel and common electrodes positioned on opposing sides of the capsule layer. Text legibility on EPDs is compromised by limited grayscale resolution and particle diffusion, resulting in blurred strokes and fragmented character structures. While waveform-based corrections offer partial mitigation, they inadequately preserve fine typographic details critical for educational and commercial display applications. The researchers focused on designing a systematic approach to driving waveforms. In [14], a dynamic multiwaveform invocation and local time-division refresh (LTDR) technology that includes fast-driving waveforms was proposed to reduce the driving time and achieve low flicker and smooth video display on EPDs. The authors in [16] investigated the viscous properties of the suspension, characterized the response latency of the device, and subsequently developed a novel driving waveform predicated on the response latency analysis of EPDs. A design rule for driving waveforms was proposed in [17] to mitigate ghost image artifacts on EPDs with fewer flickers, shorter driving time, and better human perception. In [15], an EPD driving waveform is introduced to utilize the black state as the reference gray level for further reducing driving duration.

However, due to substantial differences in volume, density, and charge between black and white particles in electrophoretic microcapsules, driving waveform design easily forms ghost images that seriously degrade high-resolution image performance.

### 2.2. AI Technology for Enhancing Image Resolution on EPDs

Driven by the rapid evolution of AI technology, image restoration tasks such as image super-resolution, ghost image removal, denoising and compression aim to reconstruct high-quality images from low-quality ones, which have the capability of better enhancing the image clarity and generating high-quality images on EPDs. CNNs are often used to enhance image quality on EPDs, which can clearly recognize image features, ghost images, and so forth to generate corresponding waveforms of lookup tables (LUTs) for multi-grayscale and achieve more accurate grayscales. In [18], the authors proposed employing a CNN to automatically detect ghost images, leveraging LUTs for ghost image suppression and accurate grayscale reconstruction through optimized waveform design. The authors in [20] introduced a unified end-to-end architecture for text spotting, defined as the joint task of localizing and recognizing text in natural scene imagery, alongside text-driven image retrieval. Their approach leverages a region proposal mechanism for detection and deep CNNs for subsequent recognition. The self-attention mechanism of Transformer architecture can capture global interactive information and performs particularly well in high-level visual tasks.

However, the local receptive field and nonadaptive content limitations of convolution operators in CNNs still exist: the same convolution kernel lacks specificity when applied to different image regions, and it is difficult to model long-range dependencies by relying only on fixed-sized neighborhoods. The self-attention mechanism of Transformer architecture used directly for image restoration has faced challenges, requiring massive parameters and data to achieve good results. The Swin Transformer combines local window attention with a shifted window strategy, which introduces the global dependency modeling capability of the Transformer while retaining the efficient local computation of CNNs. To balance performance and efficiency improvements, based on the Swin Transformer, researchers have proposed the Image Restoration Using Swin Transformer (SwinIR) model, which is applied to the field of image restoration [21].

### 2.3. Meta-Heuristic Optimization Algorithm Applied for Improving AI Models

Currently, existing works apply some meta-heuristic optimization algorithms to further optimize AI models for achieving better performance. The authors in [22] proposed a joint algorithm integrating Modified Particle Swarm Optimization (SMCPSO) with Fast Super-resolution Convolutional Neural Networks (FSRCNN) to enhance the efficiency and accuracy of AI models. In [23], the authors introduced variant Particle Swarm Optimization (PSO) methods for fine-tuning the hyperparameters of pre-trained deep learning models, thereby improving the exploratory capabilities of CNNs. In [24], the authors presented a novel approach that combines CNN with Grey Wolf Optimization (GWO) to address challenges encountered in image super-resolution. The author in [25] proposed a GWO-based face image super-resolution (FSR) algorithm, named FSR-GWO, to mitigate the low-resolution problem more effectively.

However, abundant meta-heuristic optimization algorithms are prone to premature convergence to local optima or require extensive computational time to reach global optima, necessitating further refinement for improved optimization of AI models.

In summary, existing learning-based EPD studies primarily focus on artifact detection or classification, rather than direct text image restoration. Moreover, most methods are designed to cooperate with waveform optimization, serving as auxiliary modules rather than standalone enhancement solutions. As a result, they still depend on hardware intervention and cannot be directly applied to legacy or fixed-waveform EPD devices.

## 3. Methodology

**Figure 1:**
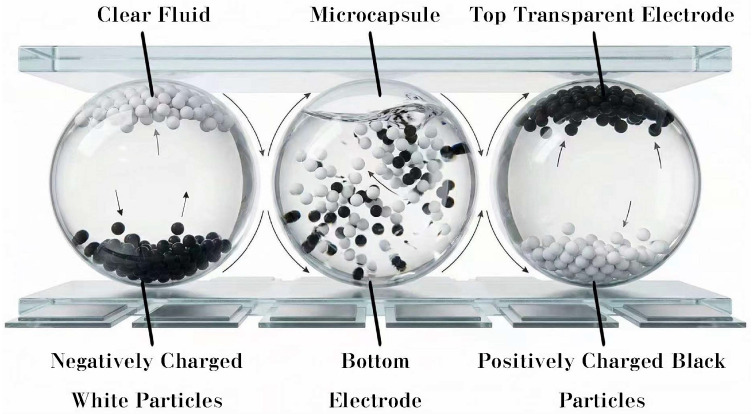
The structure of a microcapsule EPD.

As depicted in Figure 1, the EPDs operate on the principle of ambient light reflection modulated by electrophoretic migration of dichromatic or polychromatic charged particles. Through the application of bipolar voltage waveforms with programmable temporal characteristics, pigment particles of varying optical properties are electrophoretically driven to specified spatial locations within encapsulated suspensions, facilitating bistable, high-resolution text image formation on EPDs.

To achieve better performance on the super-resolution and recognition of text images on EPDs, this paper proposes a novel IWOA-MFA-SwinIR model in this section to enhance text readability at the text image level without modifying display hardware or driving waveform, as a new modular and configurable SwinIR model containing three attention mechanisms—channel attention mechanism, spatial attention mechanism, and gated attention mechanism—and a multi-scale fused attention module.

**Figure 2:**
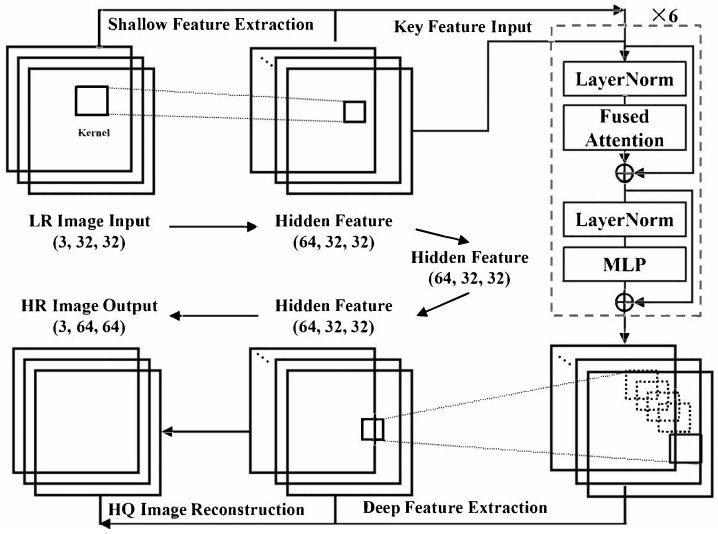
Overall framework of the our proposed IWOA-MFA-SwinIR model improved by incorporating self-attention.

The overall process of our proposed IWOA-MFA-SwinIR model is shown in Figure 2. According to the three-stage structure (shallow feature extraction, deep feature extraction, and high-quality image reconstruction) of the original SwinIR model, our proposed model takes an LR image (3, 32, 32) as input and progressively transforms it through these stages to produce an HR image output (3, 64, 64). Specifically, the shallow feature extraction module first extracts low-level features from the LR image input (3, 32, 32), followed by the deep feature extraction stage that captures high-level semantic information through embedded attention modules and fusion mechanisms. Finally, the high-quality image reconstruction stage restores the high-frequency details and generates the final HR image output (3, 64, 64). By embedding attention modules and fusion mechanisms, our proposed IWOA-MFA-SwinIR model is capable of achieving precise capture of text features and suppression of redundant information while maintaining its lightweight characteristics.

In Figure 2, “Fused Attention” in our proposed IWOA-MFA-SwinIR model represents the adopted fused self-attention module, which utilizes fusion methods from three above-mentioned attention mechanisms.

Now, the original SwinIR model and its preliminaries are briefly introduced in the following figure.

### 3.1. Original SwinIR Model

The original SwinIR model is structured into three modules: shallow feature extraction, deep feature extraction, and high-quality image reconstruction. The shallow feature extraction module employs a single convolutional layer to capture initial feature representations, which are subsequently propagated directly to the reconstruction module via long skip connections, thereby effectively preserving low-frequency information. The deep feature extraction module consists of multiple Residual Swin Transformer Blocks (RSTBs), wherein each block integrates multiple Swin Transformer layers to perform localized self-attention computations and facilitate cross-window information exchange. Additionally, a convolutional layer is appended at the end of each block to augment feature expressiveness. Simultaneously, residual connections run through both the interior and inter-block connections, providing shortcut paths for feature aggregation. After multi-level feature extraction, the features extracted from the shallow and deep layers are fused in the reconstruction module to output restored high-quality images. For super-resolution tasks that require resolution enhancement, the reconstruction module employs subpixel convolution for upsampling; for tasks that do not require size enlargement, such as denoising and compression artifact removal, convolutional layers are directly used for reconstruction output. Additionally, during SwinIR training, a residual learning strategy is introduced to only learn the differences between input and output images, stabilizing the optimization process and improving model convergence speed and performance.

Compared to traditional CNN-based models, the SwinIR model demonstrates multiple technical advantages through its Transformer architecture. Firstly, the self-attention mechanism adaptively allocates attention weights based on image content, achieving a dynamic convolution effect related to image content. This means performing spatially variable convolution operations for different positions, thereby better handling local content during the restoration process. Secondly, the shifted window strategy enables the model to capture long-distance dependencies based on local attention computation, effectively modeling the global range of image correlations and compensating for the lack of global perception ability in convolutional networks. Lastly, thanks to the efficient window attention design and residual architecture, SwinIR achieves excellent performance while maintaining low model complexity (number of parameters), that is, achieving higher reconstruction accuracy with fewer parameters. This balance between performance and efficiency makes SwinIR stand out among similar methods.

### 3.2. Principle of Key Improvement Modules

In this section, our study proposes a new IWOA-MFA-SwinIR model to enhance text readability at the text image level without modifying display hardware or driving waveforms.

#### 3.2.1. Channel Attention (CA) Module

To highlight feature channels related to text semantics and suppress invalid background information, our study adopts the channel attention module in this section to perform statistical learning on the channel dimension of the feature map by adaptively adjusting the weights of different channels. Its core purpose is to capture global information of channels through average pooling and max pooling, and learn the weight distribution through a fully connected layer. The expressions of the channel attention module are listed as follows:

(1) $$M_{c}\left(F\right)=\sigma \left(W_{w}\delta \left(W_{1}\cdot \mathrm{AvgPool}\left(F\right)\right)+W_{2}\delta \left(W_{1}\mathrm{MaxPool}\left(F\right)\right)\right),$$

(2) $$F_{CA}=F⨂M_{C}\left(F\right),$$

where $F\in R^{C\times H\times W}$ is the input feature map, with *C* representing the number of channels and *H*, *W* indicating the spatial dimensions; AvgPool(·) and MaxPool(·) correspond to global average pooling and global max pooling operations, respectively; $W_{1}\in R^{C/r\times C}$ and $W_{2}\in R^{C\times C/r}$ are the fully connected layer weight matrices; *r* is the dimensionality reduction coefficient; δ and σ denote the ReLU and Sigmoid activation functions, respectively; ⨂ represents element-wise multiplication, and $F_{CA}$ represents the output feature map after channel attention weighting.

#### 3.2.2. Spatial Attention (SA) Module

To suppress background noise interference on EPDs, this section deploys the spatial attention module that focuses on the spatial dimension of the feature map by learning the spatial position weights of text regions to enhance the feature representation of key structures, including character outlines and strokes on EPDs. Its core is to perform convolutional learning on the statistical information of the channel dimension to generate a spatial attention map. The mathematical equation of the spatial attention module can be expressed by

(3) $$M_{S}\left(F\right)=\sigma \left(\mathrm{Conv}(\mathrm{cat}(\mathrm{AvgPool}(F\right),\mathrm{MaxPool}\left(F\right)))),$$

(4) $$F_{SA}=F⨂M_{S}\left(F\right),$$

where cat(·,·) denotes the channel dimension concatenation operation, which concatenates the average pooling and max pooling results into a feature map of 2×H×W; Conv(·) denotes the 3×3 convolution operation to maintain the spatial dimensions as is; is the generated spatial attention map; and $M_{S}\left(F\right)\in R^{1\times H\times W}$ is the output feature after spatial attention weighting.

#### 3.2.3. Gated Attention (GA) Module

**Figure 3:**
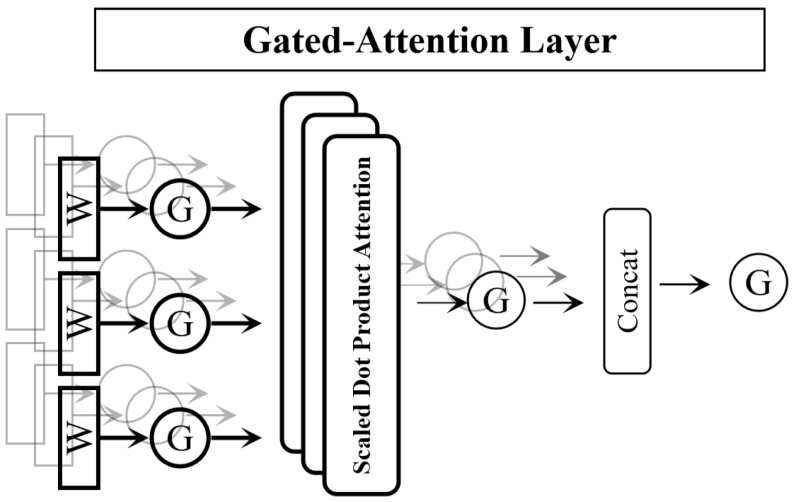
Architecture of the GA mechanism.

To solve the “attention sinking” problem of traditional attention mechanisms, based on the SwiGLU activation mechanism, our study applies the gated attention module in this section to achieve nonlinear sparse learning of features through a gating mechanism and maintain efficient computation. This module that consists of a gated network and a value network ensures information integrity through residual connections, of which the expression is as follows:

(5) $$\mathrm{gate}=\sigma ({\mathrm{Conv}}_{2}\left(\delta_{1}\left({\mathrm{Conv}}_{1}\left(F\right)\right)\right)),$$

(6) $$\mathrm{value}=\delta ({\mathrm{Conv}}_{4}\left(\delta_{1}\left({\mathrm{Conv}}_{3}\left(F\right)\right)\right)),$$

(7) $$F_{G4}=F+\mathrm{gate}⨂\mathrm{value},$$

where Conv_1_ and Conv_3_$\in R^{(C/r)\times C\times 1\times 1}$ are the down-dimensional convolution kernels; Conv_2_ and Conv_4_$\in R^{C\times (C/r)\times 1\times 1}$ are the up-dimensional convolution kernels; $\delta_{1}$ represents the SiLU activation function (activated by the Swin model), and $\delta_{2}$ represents the GELU activation function; gate $\in R^{C\times H\times W}$ is the gated weight map that achieves feature sparsification; $F_{GA}$ is the gated attention output feature, and the residual connection ensures that the original information is not lost. The detailed gated module structure is shown in Figure 3.

#### 3.2.4. Multi-Scale Fused Attention (MFA) Module

**Figure 4:**
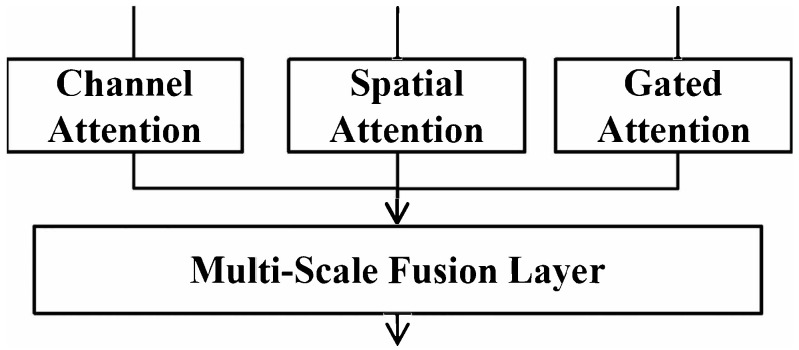
Architecture of multi-scale fusion layer driven by the MFA mechanism.

In this section, the multi-scale fused attention (MFA) module is utilized to perform weighted fusion on the output of the above-mentioned three attention mechanisms in the deep feature extraction stage, which fully uses feature information from different levels—shallow texture features and deep semantic features—and adaptively adjusts the contribution of each scale of features through learnable weights, as shown in Figure 4. The mathematical equation of the MFA module is expressed by

(8) $$F_{MSF}=F_{shadow}+\sum_{i=1}^{N}w_{i}F_{i},$$

where $F_{i}\in R^{C\times H\times W}$ represents the output feature of the *i*-th Residual Swin Transformer Block, $F_{shadow}$ is the shallow output feature, and *N* is the total number of Transformer layers; $w_{i}\in R$ is the learnable fusion weight that satisfies $\sum_{i=1}^{N}w_{i}=1$; and $F_{MSF}$ is the output feature after multi-scale fusion.

In summary, the section presents the MFA-SwinIR model, which enhances the original SwinIR model through the integration of channel, spatial, and gated attention mechanisms coupled with multi-scale feature fusion to enable precise text edge modeling and background noise suppression. Furthermore, it effectively overcomes the super-resolution and recognition inadequacies of general-purpose text image enhancement models on EPDs.

### 3.3. Improved Whale Optimization Algorithm (IWOA)

**Figure 5:**
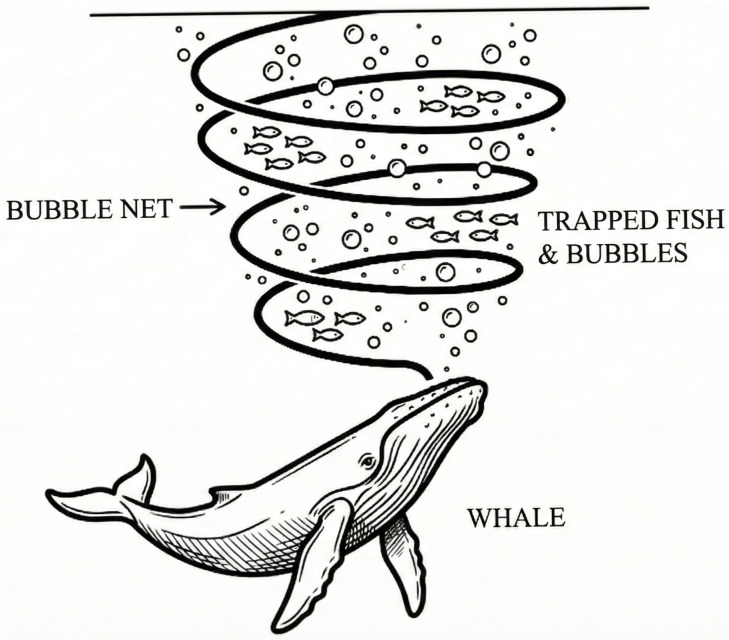
Feeding behavior of a humpback whale.

The WOA proposed in [26], a meta-heuristic optimization algorithm mimicking humpback whales’ bubble-net feeding strategy during foraging, has been widely used in various optimization domains to emulate their sophisticated predatory behavior, as shown in Figure 5. The bubble-net feeding strategy is utilized by humpback whales to capture aggregations of krill and small fish and encompasses three sequential phases: prey encirclement, bubble-net assault, and foraging search. However, the traditional WOA manifests intrinsic deficiencies, notably the proneness to premature convergence toward local optima and suboptimal global exploration efficiency, which may compromise the stringent temporal constraints requisite for performance optimization tasks. To ameliorate these limitations, the present study proposes a new IWOA meticulously tailored for the fine-tuning of critical hyperparameters inherent to the aforementioned MFA-SwinIR architecture.

This section introduces the comprehensive framework of the IWOA that comprises three core improvements—Sobol sequence-based population initialization, nonlinear convergence factor adjustment, and enhanced boundary handling mechanism.

The PSNR value serves as the objective function to quantify the fitness landscape, where the optimization objective relates to maximizing the PSNR values. The algorithm initiates with a stochastic population comprising *N* candidate solutions (whales) distributed across a *d*-dimensional decision space, with each whale’s position $X_{i}=\left(X_{i}^{1},X_{i}^{2},\ldots ,X_{i}^{d}\right)$ representing a prospective PSNR value configuration.

#### 3.3.1. Sobol Sequence-Based Population Initialization

The traditional WOA typically employs a stochastic initialization mechanism for population generation, potentially yielding insufficient solution space coverage and limited population diversity. Such a stochastic initialization mechanism may result in sparse optimal solutions distant from the initialized population, thereby increasing susceptibility to local optima entrapment. This algorithm demonstrates suboptimality for achieving superior PSNR, SSIM, and CRA in the super-resolution and recognition of text images on EPDs.

In this section, a Sobol sequence is employed to initialize whale positions with uniform distribution within the range of [0, 1], which can enhance population diversity and improve the algorithm’s exploratory capabilities.

(9) $$X_{i}=X_{min}+\eta \left(X_{max}-X_{min}\right),$$

where $X_{max}$ and $X_{min}$ denote the lower and upper bounds of the position, respectively, and η∈[0,1] is a quasi-random number generated via the Sobol sequence.

#### 3.3.2. Nonlinear Convergence Factor Adjustment

In the traditional WOA, the parameter $\vec{A}$, governing the stride of positional vectors through $\vec{A}\cdot \vec{D}$, critically modulates the trade-off between global exploration and local exploitation in WOA. Its magnitude depends on the convergence factor $\vec{a}$, where elevated values of $\vec{A}$ enhance global search capabilities to avoid entrapment of local optima, while diminished values intensify local exploitation to accelerate convergence. However, the linear decay of $\vec{a}$ with respect to iteration count in conventional WOA may impede convergence velocity, thereby compromising the expeditious forecasting requirements for the super-resolution and recognition of text images on EPDs. To address these limitations—specifically, the propensity for premature convergence and protracted global optimization—this study proposes an IWOA incorporating a nonlinear adjustment mechanism for $\vec{a}$. This adaptive strategy dynamically balances exploration and exploitation throughout the optimization trajectory, formulated as follows:

(10) $$a=\left(2-\frac{2k}{K_{max}}\right)\left(1-\frac{k^{3}}{K_{max}^{3}}\right),$$

where *k* and $K_{max}$ denote the current and maximum iteration numbers, respectively. The nonlinear convergence factor $\vec{a}$ scales the parameter $\vec{A}$ adaptively: larger values in early iterations promote global exploration, while smaller values in later iterations facilitate local exploitation. This nonlinear convergence factor adjustment efficiently minimizes the PSNR values, yielding enhanced performance in terms of PSNR, SSIM, and CRA values for text image super-resolution and recognition on EPDs, with accelerated convergence.

#### 3.3.3. Enhanced Boundary Handling Mechanism

In the traditional WOA, out-of-bound whales are typically eliminated or retracted to boundary constraints, which compromises population diversity and induces boundary aggregation that impairs convergence efficiency. To address this limitation, a novel enhanced boundary handling mechanism is proposed. This mechanism preserves population diversity, enhances solution quality post-update, and accelerates convergence toward the current global optimum. Furthermore, this mechanism demonstrably facilitates PSNR value maximization, thus expediting the attainment of optimal solutions, formulated as follows:

(11) $$\begin{array}{cc} X_{inew} & =X_{i}\times H,\;\;\;\;\;\;\;\;\mathrm{if}\;\;X\geq X_{up}\;\mathrm{or}\;\;X\leq X_{low},\\ H & =L\times rand(-0.5,0.5)/D, \end{array}$$

where *L* denotes the current Euclidean distance between the best-positioned whale and its closest neighbor, and *D* represents the dimensionality of the search space.

## 4. Establishment of IWOA-MFA-SwinIR Model

In this section, our study proposes a novel IWOA-MFA-SwinIR model to enhance text readability at the text image level without modifying display hardware or driving waveforms.

IWOA is utilized to improve the parameter search process of the MFA-SwinIR model. This paper employs the IWOA to refine four critical hyperparameters of the SwinIR model, which includes embedding dimension (*d*), attention head count (*h*), learning rate (lr), and dimensionality reduction coefficient (*r*).

The specific process of our proposed IWOA-MFA-SwinIR model is as follows:

**Step 1. Initialization.** Initialize IWOA population size *N*, maximum iterations *T*, initial $\vec{a}$, and configure four critical hyperparameters to be optimized and their search ranges: embedding dimension *d*, attention head count *h*, learning rate lr (logarithmic distribution), and dimensionality reduction coefficient *r*;

**Step 2. Definition of the fitness function.** Utilize the PSNR value of the model on the TextZoom validation set as the fitness function f(X). Higher values indicate superior parameter combinations. The formula of the fitness function f(X) is as follows:

(12) f(X)=PSNR(SR(X),HR);

where SR(X) is the super-resolution and recognition of text images generated by the model under the parameter *X* combination, and HR is the real high-resolution text image.

**Step 3. Application of the IWOA.** Calculate each whale’ initial position and fitness in every iteration and select the current optimal individual ${\vec{X}}_{new}$ by using the IWOA comprising three improvements: Sobol sequence-based population initialization, nonlinear convergence factor adjustment, and enhanced boundary handling mechanism;

**Step 4. Iterative optimization.** Update the coefficients $\vec{a}$, $\vec{A}$, AND $\vec{C}$ and the random number *p*, and select the contraction envelope or spiral update strategy to update the individual positions based on *p*;

**Step 5. Select THE best optimal parameter for the MFA-SwinIR model.** Determine whether the maximum iteration count has been reached or the fitness has converged (with a fitness improvement of less than $1\times 10^{-4}$ for 5 consecutive generations). If either condition is met, stop iterating and output the optimal parameter combination; otherwise, return to **Step 3**.

**Step 6. Verification of optimal parameters in the MFA-SwinIR model.** Substitute the optimal parameters obtained from THE IWOA search into the MFA-SwinIR model, and verify the model performance on the test set.

**Figure 6:**
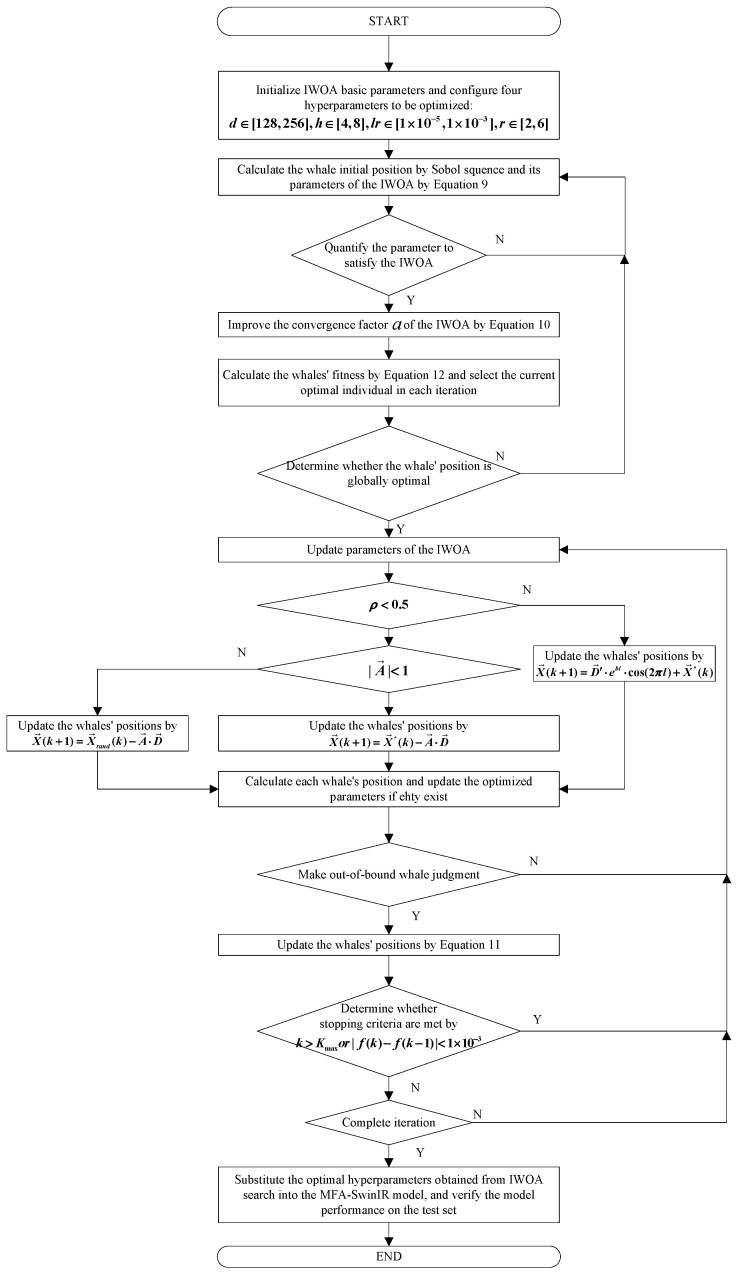
The flowchart of our proposed IWOA-MFA-SwinIR model on EPDs.

The flowchart of our proposed IWOA-MFA-SwinIR model on EPDs is shown in Figure 6.

## 5. Experiments

To build a new EPD dataset for learning and evaluating real-world displayS through e-paper, we collect the low-resolution (LR) text images and design a novel EPD system to form the high-resolution (HR) text images for better enhancing text readability at the text image level without modifying display hardware or driving waveform. Our designed system has the capability of performing to generate the LR-HR pairs of the same text content on EPDs, which includes two parts: an LR text image recognition system and an HR text image generation system.

**Figure 7:**
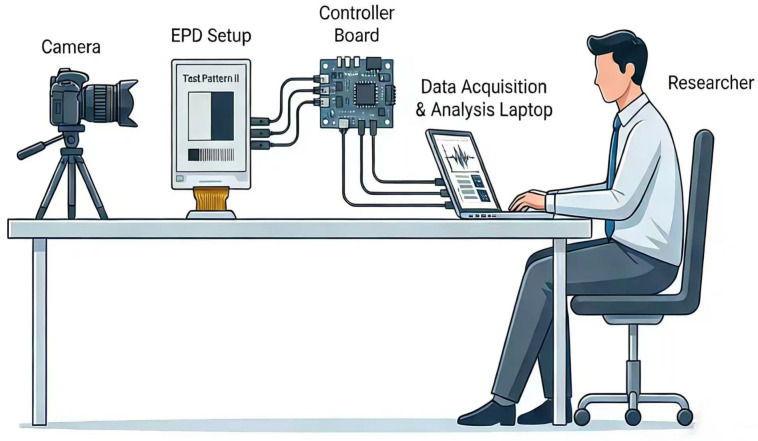
The experimental scenario for EPD performance evaluation.

Figure 7 shows the experimental scenario that comprises four integrated components: a high-resolution camera mounted on a tripod for image capture, an EPD panel displaying test patterns, a controller board for device driving and signal processing, and a data acquisition and analysis laptop operated by a researcher. The camera captures visual data from the EPD screen, which is driven by the controller board and connected to the laptop for real-time monitoring, data collection, and subsequent analysis.

### 5.1. Experimental Configuration

In this section, our study introduces a series of experimental configurations, which contain the dataset configuration, the optimized parameter configuration, and the model configuration for EPDs. Moreover, evaluation metrics as the measurement criteria of the generated HR text image are also given in the following.

#### 5.1.1. Dataset Configuration

**Figure 8:**
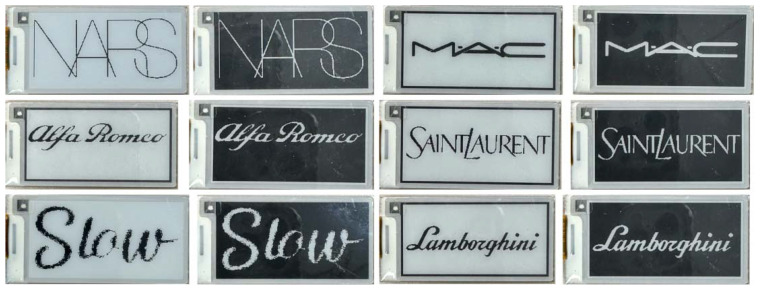
Preview of partial EPDs dataset.

In this section, we elaborate on the datasets employed for training and evaluation. The training set comprises two distinct sources:

(a) The TextZoom dataset is first built from two real paired LR–HR datasets: RealSR [27] and SRRAW [28] that capture the same scene at multiple camera focal lengths. Secondly, we annotate word bounding boxes on the images taken with the largest focal length in each group. Thirdly, we annotate crop-corresponding text regions from the other focal-length images using the same rectangle. Fourthly, all LR text images are up-sampled to 64 × 16 and HR images to 128 × 32 before evaluation/training pipelines to avoid introducing down-sampling degradation. They further create a simple binary text mask by thresholding based on the mean grayscale of the RGB image and concatenate it with RGB as a 4-channel input (RGBM). Finally, the dataset is split into easy/medium/hard subsets by difficulty levels.

(b) The EPDs dataset, specifically designed for electronic paper applications featuring experiments conducted under both black-text-on-white-background and white-text-on-black-background configurations, and representative samples from the EPDs dataset are presented in Figure 8. The model’s performance exhibits dependency on the quality of initial LR text images captured by the recognition system, necessitating selective LR sample generation. Detailed specifications of the data acquisition parameters and imaging conditions can be found in [29]. We have adopted GoodDisplay’s product (No. GDEY0213B74) for our experiment [30], which uses the Image2LCD software to achieve text images on EPDs. Text image samples on EPDs were captured utilizing a digital imaging system under regulated indoor lighting environments, employing diffuse light sources to eliminate specular reflections and shadow artifacts that may degrade the Optical Character Recognition (OCR) system. All acquired images were resampled to a uniform spatial dimension of 250 × 122 pixels to facilitate consistent feature extraction and network input standardization.

#### 5.1.2. Optimized Parameter Configuration by IWOA

**i. Initialization of IWOA Basic Parameters.** This section initializes IWOA population size N=30, maximum iterations T=10, initial $\vec{a}=2$, and configures four critical hyperparameters to be optimized and their search ranges: embedding dimension d∈[128,256] (step size 32), attention head count h∈[4,8] (step size 2), learning rate $lr\in [1\times 10^{-5},1\times 10^{-3}]$ (logarithmic distribution), and dimensionality reduction coefficient r∈[2,6].

**ii. Determination of IWOA Final Optimal Hyperparameters.** Through IWOA iterative optimization, the combination of the final optimal hyperparameters is determined as follows: embedding dimension d=180, attention head count h=6, learning rate $lr=8.2\times 10^{-4}$, and dimensionality reduction coefficient r=4. This parameter combination achieves an optimal balance between visual quality and semantic recognition accuracy while ensuring model lightweightness.

#### 5.1.3. Model Configuration

This experiment is divided into ablation studies and comparative experiments. Among them, the eight ablation configurations are SwinIR (Baseline), Channel Attention (Only CA), Spatial Attention (Only SA), Gated Attention (Only GA), +CA+SA, +CA+GA, +SA+GA, and IWOA-MFA-SwinIR (Ours). The comparative experiment compares six classic and convenient models, which include TSRN [31], SRCNN [32], VDSR [33], SRResNet [34], ESRGAN [35], and EDSR [36]. The hardware and environment for our experiment are an NVIDIA RTX 5090 (32 GB), the PyTorch framework, Python version 3.12, and a pre-trained CRNN model as the recognizer.

#### 5.1.4. Evaluation Metrics

There are three evaluation metrics for super-resolution tasks to make performance evaluations in this section, which are divided into visual quality metrics and semantic quality metrics. The PSNR and the SSIM, as two visual quality metrics, are often used to measure the pixel-level similarity between the generated high-resolution text images and the real text images. The PSNR, serving as a visual quality metric and the primary optimization objective, is used to measure the pixel-level error of the image, with a higher value indicating better image quality, of which the formula is as follows:

(13) $$\mathrm{PSNR}=10\cdot log_{10}\left(\frac{MAX_{I}^{2}}{MSE(I_{HR},ISR)}\right),$$

The SSIM, adopted herein as the secondary fidelity measure and a semantic quality metric, is applied to measure the structural similarity of images from three dimensions: brightness, contrast, and structure. Its value is in the range of [0, 1], with a value closer to 1 indicating greater similarity. Its formula is the following:

(14) $$\mathrm{SSIM}\left(x,y\right)=\frac{\left(2\mu_{x}\mu_{y}+C_{1}\right)\left(2\sigma_{xy}+C_{2}\right)}{\left(\mu_{x}^{2}+\mu_{y}^{2}+C_{1}\right)\left(\sigma_{x}^{2}+\sigma_{y}^{2}+C_{2}\right)},$$

where $x=I_{HR}$, $\;y=I_{SR}$, $\mu_{x},\;\mu_{y}$ are, respectively, the local mean of *x* and *y*, $\sigma_{x}^{2},\;\sigma_{y}^{2}$ are, respectively, the local variance of *x* and *y*, and $\sigma_{xy}$ is the local covariance of both *x* and *y*. $C_{1}$ and $C_{2}$ are constants, which are often considered as $C_{1}={\left(k_{1}\times MAX_{I}\right)}^{2}$ and $C_{2}={\left(k_{2}\times MAX_{I}\right)}^{2}$, where $k_{1}=0.01$, $k_{2}=0.03$.

Furthermore, a semantic quality metric is used for measuring the CRA values (acting as an auxiliary task-specific validator), which performs the OCR system on both HR and SR text images and calculates the matching rate of the recognized characters. The OCR system refers to computer vision technology that detects characters in e-paper through electronic devices and converts them into editable text using image processing and pattern recognition techniques. Its pipeline comprises core modules including image preprocessing, text detection, and character recognition to enhance recognition accuracy on EPDs. The CRA’s expression can be described by

(15) $$\mathrm{CRA}=\frac{\mathrm{Number}\;\mathrm{of}\;\mathrm{Matched}\;\mathrm{Characters}}{\mathrm{Total}\;\mathrm{Number}\;\mathrm{of}\;\mathrm{Characters}\;\mathrm{in}\;I_{HR}}\times 100\%,$$

The hierarchical ranking for all metrics is PSNR > SSIM > CRA, reflecting the trade-off between mathematical optimization tractability and end-to-end application performance, where the WOA-driven PSNR maximization implicitly facilitates improved SSIM and CRA values through enhanced image fidelity.

Additionally, Frames Per Second (FPS) and Floating-point Operations (FLOPs) are widely used to evaluate the computing power of computer hardware.

FLOPs (Floating-point Operations): FLOPs refer to the number of floating-point arithmetic operations required to execute a given computational task, serving as a quantitative measure of computational complexity (temporal complexity). In the context of neural network evaluation, FLOPs are widely adopted as an indirect proxy for model inference speed and computational efficiency, where lower FLOPs typically indicate reduced computational overhead and faster theoretical execution, though actual latency may vary depending on hardware implementation and optimization strategies.

FPS (Frames Per Second): FPS denotes the frame rate, defined as the number of images processed per unit time (typically one second), or equivalently, the inverse of the processing time required for a single image. This metric provides a direct empirical assessment of detection or reconstruction speed in real-time applications, where higher FPS values (or equivalently, shorter per-image processing intervals) indicate superior temporal throughput and enhanced practical deployability in latency-sensitive scenarios.

### 5.2. Ablation Experiment

#### 5.2.1. Design of Ablation Experiment

The ablation experiment aims to verify the effectiveness of various attention modules and their combinations. Three attention mechanisms are separately incorporated into the model for testing. A total of eight sets of experiments are set up, with specific configurations shown in Table 1.

#### 5.2.2. Ablation Experimental Results of TextZoom Dataset

As listed in Table 2, the ablation experimental results of both the super-resolution and recognition tasks on EPDs in the TextZoom test set demonstrate that our proposed IWOA-MFA-SwinIR model incorporating multiple attention mechanisms exhibits optimal performance across all difficulty levels that includes the easy level, the medium level, and the hard level. In the scenario of the easy level, our proposed IWOA-MFA-SwinIR model achieved a PSNR value of 24.406, a SSIM value of 0.8837, and a CRA value of 89.81%. In the scenarios of the medium and hard levels, its PSNR values reach 20.7935 and 21.0668, its SSIM values attain 0.6708 and 0.7509, and CRA values are 72% and 69.47%, respectively. In particular, all indicators demonstrate the superiority of our proposed IWOA-MFA-SwinIR model over both the baseline SwinIR model and other single or dual attention mechanism combination models on EPDs.

Moreover, as shown in Table 3, the ablation experimental results demonstrate that our proposed IWOA-MFA-SwinIR model strikes a favorable balance between recognition accuracy and computer hardware efficiency, preserving high precision with minimal sacrifice in computational speed.

**Figure 9:**
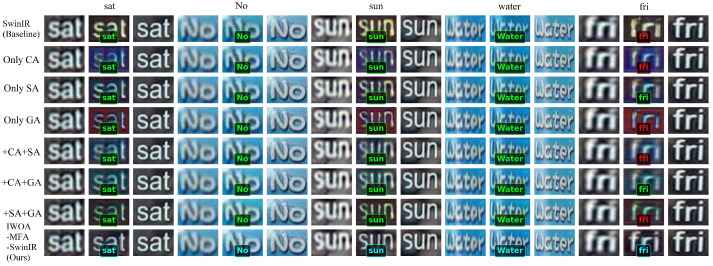
Ablation experimental performance of different modules on the test dataset.

Notably, the visual reconstruction results in Figure 9 further validate the conclusion that our proposed IWOA-MFA-SwinIR model exhibits the sharpest reconstruction edges for text characters, such as “sat”, “No”, “sun”, “water”, and “fri”, with the most accurate color reproduction and higher accuracy in recognition box annotation. The red color in Figure 9 indicates model recognition error, while the blue and green colors indicate that the model correctly recognizes the text content. In comparison, the other models that only bring in a single attention mechanism, such as Only CA, Only SA, Only GA, or a combination of dual attention mechanisms, show limited performance improvement in the medium and hard levels, although performing well in the easy level. Detailed ablation experimental results of the super-resolution and the text recognition on EPDs are shown in Figure 9.

**Figure 10:**
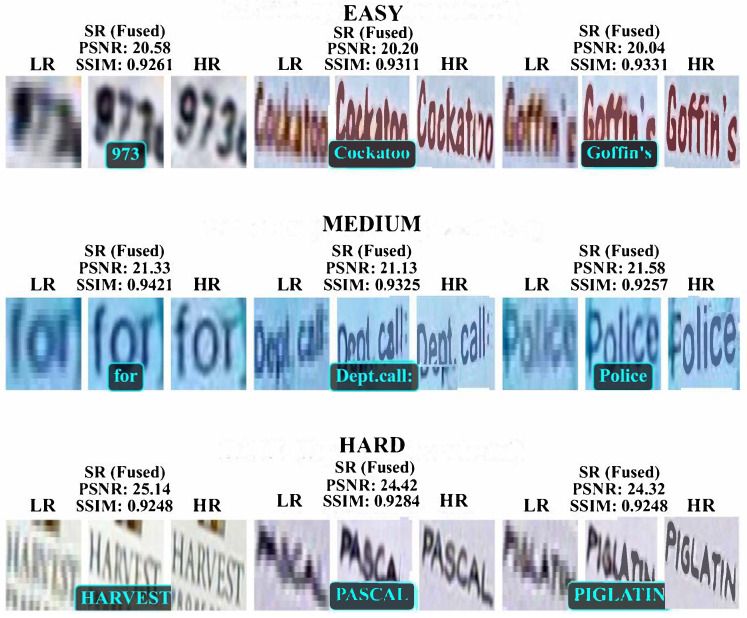
Super resolution performance of our proposed IWOA-MFA-SwinIR model at different difficulty levels.

From the visual effects depicted in Figure 10, our proposed IWOA-MFA-SwinIR model demonstrates its capabilities as follows:

(a) In the easy level, our proposed IWOA-MFA-SwinIR model clearly restores characters such as “973”, “Cockatoo”, and “Goffin’s” with sharp edges, achieving high visual consistency with high-resolution reference images. The corresponding PSNR value is approximately 20.04–20.58, and the SSIM value is around 0.9261–0.9331.

(b) In the medium level, our proposed IWOA-MFA-SwinIR model maintains good clarity in the reconstruction of words like “for”, “Dept.call”, and “Police”. The PSNR value increases to 21.13–21.58, and the SSIM value is around 0.9257–0.9421, showcasing robustness in moderately complex scenes.

(c) In the hard level, our proposed IWOA-MFA-SwinIR model still accurately identifies and reconstructs complex words, such as “HARVEST”, “PASCAL”, and “PIGLATIN”, despite significant increases in background interference and character blurring. The PSNR value further rises from 24.33 to 25.13, and the SSIM value remains stable at [0.9248,0.9284], indicating that the fused model maintains superior reconstruction capabilities even in challenging e-paper scenarios.

In summary, as the difficulty level increases from easy to hard, both the PSNR value and the SSIM value of our proposed IWOA-MFA-SwinIR model remain at high levels. This indicates that the fusion model not only maintains high visual quality in simple scenarios but also effectively enhances the clarity and fidelity of images in complex scenarios in the super-resolution and recognition of text images on EPDs.

### 5.3. Test Results of EPDs Dataset

**Figure 11:**
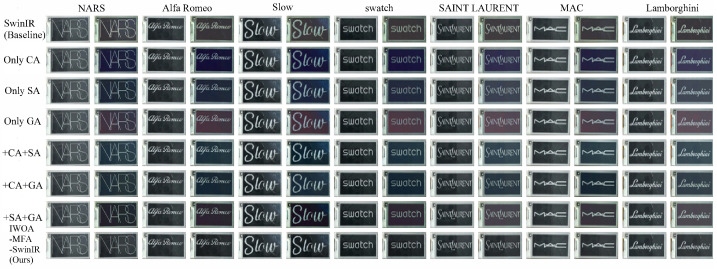
Super-resolution effect of different models on EPDs with black background.

**Figure 12:**
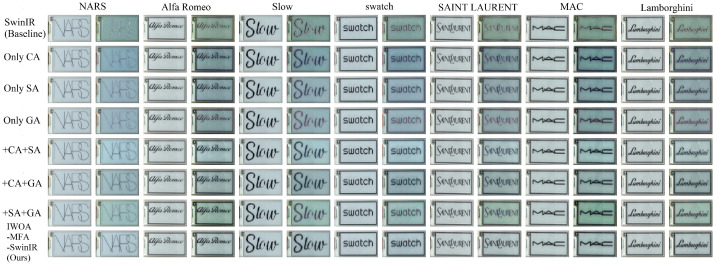
Super-resolution effect of different models on EPDs with white background.

In this section, our study tests the proposed IWOA-MFA-SwinIR model on EPDs with both black and white backgrounds. The image super-resolution results in the two backgrounds are plotted as shown below in Figure 11 and Figure 12. Clearly, Black backgrounds, shown in Figure 11, highlight performance gaps in text contrast, edge sharpness, and color rendition, while white backgrounds, shown in Figure 12, test each model’s ability to resolve light-colored text—both offering immediate visual grounds for comparison. By horizontally comparing the output effects of different models under two typical electronic paper backgrounds, we can clearly evaluate the optimization effect of various attention mechanisms and their combinations on EPDs.

From Table 4, it can be seen that all models that incorporate attention mechanisms significantly outperform the baseline SwinIR model in terms of both the average PSNR value and the average SSIM value. This indicates that various attention mechanisms enhance the reconstruction quality of e-paper images on EPDs.

Firstly, among the models that only incorporate a single attention mechanism, the “+SA” model performs the best (average PSNR is 15.8761, average SSIM is 0.8081), which is a bit superior to the “+CA” model and the “+GA” model.

Secondly, when we combine two attention mechanisms, the performances of the “+CA+SA” model and the “+CA+GA” model are further improved, with average PSNR values reaching 17.6029 and 16.7531, respectively, and average SSIMs exceeding 0.84, which have the performance superiority of two attention mechanisms over a single attention mechanism on both the super-resolution and recognition tasks.

Finally, our proposed IWOA-MFA-SwinIR model that integrates all three attention mechanisms exhibits the best optimal performance, with a PSNR value of 21.7813 and an SSIM value of 0.8646, when compared to the baseline model and other models with added partial attention mechanisms, which validates the effectiveness of multi-attention mechanism collaboration in both the super-resolution and recognition tasks on EPDs.

### 5.4. Comparative Experiment

#### 5.4.1. Design of Comparative Experiment

The comparative experiment compares our proposed IWOA-MFA-SwinIR model with early classic models: TSRN [31], SRCNN [32], VDSR [33]; and deep residual models: SRResNet [34], ESRGAN [35], and EDSR [36] were used to verify the comprehensive performance and generalization ability of the proposed model.

#### 5.4.2. Comparative Experimental Results of TextZoom Dataset

In the super-resolution and recognition of text images on EPDs, our proposed IWOA-MFA-SwinIR model is compared with six classic super-resolution models: TSRN [31], SRCNN [32], VDSR [33], SRResNet [34], ESRGAN [35], and EDSR [36]. From a quantitative perspective, this model demonstrates significant advantages across all difficulty levels, as listed in Table 5.

Moreover, as shown in Table 6, the comparative experimental results demonstrate that the FPS values of our proposed IWOA-MFA-SwinIR model are only lower than those in the SRCNN model, while the FLOPs values of our proposed model are smaller than those of all other comparison models.

### 5.5. Experiemntal Discussion

The three attention mechanisms, including “+CA”, “+SA”, and “+GA”, proposed in this paper can effectively enhance the performance of text image super-resolution models. Among them, the nonlinear sparsification characteristic of gated attention is crucial for text feature extraction. The combination of channel and gated attention (“+CA+GA”) achieves the best balance between performance and efficiency, which makes it suitable for scenarios where inference speed is critical. Our proposed IWOA-MFA-SwinIR model that integrates the three attention mechanisms achieves optimal performance, making it suitable for high-precision text restoration scenarios. Clearly, the proposed IWOA-MFA-SwinIR model shows the favorable performance on both the super-resolution and recognition tasks on EPDs over mainstream comparative models based on both the TextZoom dataset and the EPDs dataset, with notable performance improvements under special media conditions, especially in challenging scenarios, which verifies the model’s generalization ability.

## 6. Conclusions

This paper has proposed a novel IWOA-MFA-SwinIR model for the super-resolution and recognition of text images on EPDs to improve readability at the algorithmic level without hardware modification. The proposed model has first integrated channel, spatial, and gated attention mechanisms within an MFA module to enhance feature representation. Secondly, following the architecture of the SwinIR model (shallow feature extraction, deep feature extraction, and high-quality image reconstruction), these attention modules have precisely captured text features while suppressing redundant information, which has maintained computational efficiency. Thirdly, the IWOA has optimized four critical hyperparameters: embedding dimension (*d*), attention head count (*h*), learning rate (lr), and dimensionality reduction coefficient (*r*). Finally, experiments conducted on the TextZoom and EPD datasets demonstrate that our proposed IWOA-MFA-SwinIR model achieves state-of-the-art performance in both super-resolution and recognition tasks, as measured by quantitative metrics ((PSNR) and (SSIM)) and qualitative metrics (CRA), outperforming mainstream comparative models. In the ablation study, it attains a Peak Signal-to-Noise Ratio (PSNR) of 24.406, a Structural Similarity Index (SSIM) of 0.8837, and a Character Recognition Accuracy (CRA) of 89.81%. In the comparative evaluation, the proposed model consistently outperforms the second-best comparison model (ESRGAN) across three difficulty levels, yielding approximately a 1% improvement in PSNR, a 0.8% improvement in SSIM, and an 8% improvement in CRA. This confirms the proposed model’s superiority over mainstream comparative models in restoring text fidelity and improving recognition rates.

While our proposed IWOA-MFA-SwinIR model achieves superior perceptual fidelity in super-resolution and recognition of text images on EPDs, we acknowledge that real-time processing capabilities and hardware-software co-design constraints remain critical gaps for practical on-device deployment. Our current implementation prioritizes reconstruction quality over computational efficiency, with the MFA mechanisms introducing non-negligible inference overhead.

Therefore, future research would focus on three key directions:

(a) Lightweight architecture design [37], including attention mechanism pruning to reduce parameter complexity without sacrificing performance;

(b) Reinforcement learning for dynamic, context-aware adaptation at runtime for adapting inference strategies according to real-time constraints, such as latency/energy targets and content difficulty [38];

(c) Real-time benchmarking and deployment-oriented evaluation on real EPD hardware for guiding model redesign towards practical real-time usage;

(d) Hardware–software co-optimization for efficient on-device execution, exploring model quantization, and collaborative optimization with EPD driver circuits enable efficient on-device deployment.

These efforts aim to bridge the gap between algorithmic efficacy and practical deployability.

## Figures and Tables

**Table 1:** Configuration information of ablation experiment.

Experiment ID	Configuration Name	Channel Attention (CA)	Spatial Attention (SA)	Gated Attention (GA)	Core Validation Objective
E1	SwinIR (Baseline)	False	False	False	Original SwinIR performance baseline
E2	Only CA	True	False	False	Channel attention (acting alone)
E3	Only SA	False	True	False	Spatial attention (acting alone)
E4	Only GA	False	False	True	Gated attention (acting alone)
E5	+CA+SA	True	True	False	Channel + Spatial attention (collaborative effect)
E6	+CA+GA	True	False	True	Channel + Gated attention (collaborative effect)
E7	+SA+GA	False	True	True	Spatial + Gated attention (collaborative effect)
E8	IWOA-MFA-SwinIR (Ours)	True	True	True	Three attention modules (fusion effect)

**Table 2:** Quantitative comparison of ablation experiment on TextZoom test set (PSNR/SSIM/CRA).

Model	Difficulty	PSNR	SSIM	CRA (%)
SwinIR (Baseline)	Easy	21.9282	0.8161	81.63
	Medium	19.3404	0.6345	62.75
	Hard	19.4493	0.6832	61.44
Only CA	Easy	23.0178	0.828	81.32
	Medium	19.6416	0.6356	61.12
	Hard	19.8133	0.6881	61.37
Only SA	Easy	23.3613	0.8374	82.25
	Medium	19.6130	0.6477	63.81
	Hard	19.9717	0.7068	61.67
Only GA	Easy	23.4963	0.8426	82.87
	Medium	19.4713	0.6474	63.25
	Hard	19.7938	0.7051	61.74
+CA+SA	Easy	23.2712	0.828	79.35
	Medium	19.3931	0.6365	60.69
	Hard	19.6373	0.6833	60.92
+CA+GA	Easy	23.0456	0.8262	82.37
	Medium	19.7057	0.6479	62.68
	Hard	20.0128	0.7024	62.41
+SA+GA	Easy	22.8443	0.8264	81.07
	Medium	19.3773	0.6396	62.25
	Hard	19.6898	0.6914	61.67
IWOA-MFA-SwinIR (Ours)	Easy	**24.4060**	**0.8837**	**89.81**
	Medium	**20.7935**	**0.6708**	**72.00**
	Hard	**21.0668**	**0.7509**	**69.47**

The values using bold font are highlighted to present the best performance.

**Table 3:** Inference performance of ablation experiment on TextZoom test set (FPS and FLOPs(G)).

Model	FPS	FLOPs(G)
SwinIR (Baseline)	127.2449	2.7674
Only CA	108.1195	3.4878
Only SA	113.4282	3.4885
Only GA	111.6752	3.5680
+CA+SA	103.3455	3.4886
+CA+GA	100.2560	3.5689
+SA+GA	105.6886	3.5696
IWOA-MFA-SwinIR (Ours)	94.1110	3.5697

**Table 4:** Average indicators on model evaluation.

Model	Average PSNR	Average SSIM
SwinIR (Baseline)	13.9715	0.7731
Only CA	15.4653	0.8132
Only SA	15.8761	0.8081
Only GA	14.7647	0.7875
+CA+SA	17.6029	0.8414
+CA+GA	16.7531	0.8442
+SA+GA	15.7127	0.8245
IWOA-MFA-SwinIR (Ours)	**21.7813**	**0.8646**

The values using bold font are highlighted to present the best performance.

**Table 5:** Quantitative comparison of comparative experiment on TextZoom test set (PSNR/SSIM/CRA).

Model	Difficulty	PSNR	SSIM	CRA (%)
TSRN [31]	Easy	18.5110	0.7761	77.31
	Medium	17.2473	0.5944	61.12
	Hard	17.9057	0.6386	60.63
SRCNN [32]	Easy	21.8209	0.8054	78.91
	Medium	18.7500	0.6370	61.47
	Hard	19.1575	0.6801	61.50
VDSR [33]	Easy	22.4440	0.7989	78.73
	Medium	19.2862	0.6340	61.33
	Hard	19.6932	0.6748	61.22
SRResNet [34]	Easy	22.4260	0.8052	79.59
	Medium	19.4900	0.6307	62.32
	Hard	19.7389	0.6773	61.70
ESRGAN [35]	Easy	23.2214	0.8335	79.78
	Medium	19.3962	0.6400	63.53
	Hard	19.9177	0.6963	61.37
EDSR [36]	Easy	21.0443	0.7532	77.55
	Medium	17.6386	0.5757	61.93
	Hard	18.7689	0.6426	60.85
IWOA-MFA-SwinIR (Ours)	Easy	24.406	0.8837	89.81
	Medium	20.7935	0.6708	72.00
	Hard	21.0668	0.7509	69.47

**Table 6:** Inference performance of comparative experiment on TextZoom test set (FPS and FLOPs(G)).

Model	FPS	FLOPs(G)
TSRN	42.6	820
SRCNN	620.3	52
VDSR	58.4	612
SRResNET	24.7	1450
ESRGAN	19.6	3100
EDSR	21.1	2900
IWOA-MFA-SwinIR (Ours)	94.1110	3.5697

## Data Availability

The data presented in this study are included in the article. Further inquiries can be directed to the corresponding author.
